# Application of feed forward and recurrent neural networks in simulation of left ventricular mechanics

**DOI:** 10.1038/s41598-020-79191-4

**Published:** 2020-12-18

**Authors:** Yaghoub Dabiri, Alex Van der Velden, Kevin L. Sack, Jenny S. Choy, Julius M. Guccione, Ghassan S. Kassab

**Affiliations:** 1 3DT Holdings LLC, San Diego, CA USA; 2 California Medical Innovations Institute, 11107 Roselle, San Diego, CA 92121 USA; 3 SIMULIA, Dassault Systemes, Johnston, USA; 4 Department of Surgery, University of California San Francisco, San Francisco, CA USA

**Keywords:** Biomedical engineering, Cardiovascular biology

## Abstract

An understanding of left ventricle (LV) mechanics is fundamental for designing better preventive, diagnostic, and treatment strategies for improved heart function. Because of the costs of clinical and experimental studies to treat and understand heart function, respectively, in-silico models play an important role. Finite element (FE) models, which have been used to create in-silico LV models for different cardiac health and disease conditions, as well as cardiac device design, are time-consuming and require powerful computational resources, which limits their use when real-time results are needed. As an alternative, we sought to use deep learning (DL) for LV in-silico modeling. We used 80 four-chamber heart FE models for feed forward, as well as recurrent neural network (RNN) with long short-term memory (LSTM) models for LV pressure and volume. We used 120 LV-only FE models for training LV stress predictions. The active material properties of the myocardium and time were features for the LV pressure and volume training, and passive material properties and element centroid coordinates were features of the LV stress prediction models. For six test FE models, the DL error for LV volume was 1.599 ± 1.227 ml, and the error for pressure was 1.257 ± 0.488 mmHg; for 20 LV FE test examples, the mean absolute errors were, respectively, 0.179 ± 0.050 for myofiber, 0.049 ± 0.017 for cross-fiber, and 0.039 ± 0.011 kPa for shear stress. After training, the DL runtime was in the order of seconds whereas equivalent FE runtime was in the order of several hours (pressure and volume) or 20 min (stress). We conclude that using DL, LV in-silico simulations can be provided for applications requiring real-time results.

## Introduction

In the field of cardiovascular biomechanics, computational modeling has been used for clinical applications. One of the best examples is the use of computational fluid dynamics to quantify coronary flow to determine lesion severity^1–3^. Computational modeling has also been used extensively with respect to left ventricle (LV) biomechanics, to study heart failure (HF). The finite element (FE) method to simulate the mechanics of the LV in health and disease conditions. However, non-linear, 3D, time-dependent computational models are time-consuming, and cannot be performed in real-time to address clinical needs. For example, several CPU hours are needed to simulate a single cardiac cycle. As such, FE models cannot be used in applications that require real-time data about heart mechanics. Such applications include design of LV assistive devices (LVADs), design of artificial heart valves, Internet of Things (IoT) devices to monitor heart behavior, among others.

Machine learning (ML) methods can predict LV mechanics in real time. Because ML models can predict behavior of systems much faster than conventional numerical methods, these models have recently been used in cardiovascular engineering. For example, aortic, LV, and arterial wall mechanics have been analyzed using different ML algorithms^6–9^. Recently, a decision-tree algorithm was used to simulate LV mechanics and the results were in close agreement with FE data^10^.

Deep learning (DL), a subset of ML based on neural networks, has been applied successfully in cardiovascular mechanics^6^. The mechanical properties of the LV are affected by aging, disease conditions, and treatments such as LVADs. Prediction of LV function with alterations in mechanical properties can provide important information about LV conditions in a timely manner. Feed-forward DL models use data from the current time step to estimate the outputs, whereas recurrent neural networks (RNNs) use information from previous time steps to predict the outputs. To predict LV mechanics with alterations in mechanical properties, a feed-forward DL model uses mechanical properties to predict the LV mechanics, whereas an RNN could use data from past time steps as well as mechanical properties. The latter approach is based on describing the heart behavior as a sequence of events.

The goal of this study was to use DL to predict LV mechanics. We created FE models with alterations in active and passive material properties and provided the data to the DL models. Important parameters such as LV pressure, volume and endocardial stress were predicted based on input features, and the predictions were compared with FE results. We utilized both feed-forward and RNN approaches. In the feed-forward approach, we used mechanical properties, whereas in RNN, we used data from previous FE data as well as mechanical properties.

## Methods

### Computational set up

We used the FE software Abaqus (SIMULIA, Providence, RI, USA) to create in-silico models of the LV. Using a cluster with one compute node containing two Intel Xeon E5-2680 v4 processors, each of which contains 14 cores and a Dell precision T3600 workstation, we conducted two sets of FE simulations. In a four-chamber model that included both the right ventricle (RV) and LV, we obtained LV pressure and volume data. In an LV-only model, we obtained endocardial stress data. Each FE model had geometrical, material properties, and boundary condition specifications that have been described in our previous publications^4,5,11,12^. A short description is provided below.

The geometry of the four-chamber human model is from previous reports and the interested reader is referred to relevant references^12,13^. The geometry for the LV-only model was created from swine experiments. All animal data were obtained in accordance with our institutional review board, and all methods were performed in accordance with the relevant guidelines and regulations^14^. The constitutive equation was composed of a passive and an active part. The passive part describes the myocardium as a hyperplastic fiber-reinforced material, as below:

1 $${\Psi }_{dev} = \frac{a}{2b}e^{{b\left( {I_{1} - 3} \right)}} + \mathop \sum \limits_{i = f,s}^{{}} \frac{{a_{i} }}{{2b_{i} }}\left\{ {e^{{b_{i} \left( {I_{4i} - 1} \right)^{2} }} - 1} \right\} + \frac{{a_{fs} }}{{2b_{fs} }}\left\{ {e^{{b_{fs} \left( {I_{8fs} } \right)^{2} }} - 1} \right\}$$

$${\Psi }_{vol} = \frac{1}{D}\left( {\frac{{J^{2} - 1}}{2} - \ln \left( J \right)} \right)$$

where: $a$ and $b$ are isotropic stiffness of the tissue; ${a}_{f}$ and ${b}_{f}$ are tissue stiffness in the fiber direction; ${a}_{fs}$ and ${b}_{fs}$ are the stiffness due to the connection between fibers and sheet;

${I}_{1}$, ${I}_{4i}$ and ${I}_{8fs}$ are invariants, as follows:

2 $${\mathrm{I}}_{1} := \mathrm{tr}\left(\mathbf{C}\right)$$

$${\mathrm{I}}_{4\mathrm{i}}:= \mathbf{C}:\left({\mathbf{f}}_{0}\otimes {\mathbf{f}}_{0}\right)$$

$${\mathrm{I}}_{8\mathrm{ft}}:= \mathbf{C}:\mathrm{sym}\left({\mathbf{f}}_{0}\otimes {{\varvec{s}}}_{0}\right)$$

${\varvec{C}}$ is the right Cauchy–Green tensor, ${{\varvec{f}}}_{0}$ and ${{\varvec{s}}}_{0}$ are vectors that define the fiber and sheet directions, respectively; $J$ is the deformation gradient invariant; $D$ is a multiple of the Bulk Modulus $K$ ($\frac{2}{K}$).

The active part of the constitutive equation simulated the stress caused by contraction in the tissue as follows:

$$T_{0} = T_{max} \frac{{Ca_{0}^{2} }}{{Ca_{0}^{2} + ECa_{50}^{2} }}C_{t}$$

where: ${T}_{max}$ is the isometric tension at the largest sarcomere length and highest calcium concentration; ${Ca}_{0}$ is the peak intracellular calcium concentration. C_t_ is given by the following relation:

3 $$C_{t} = \frac{1}{2}\left( {1 - cos\omega } \right)$$

$$\omega =\left\{\begin{array}{c}\pi \frac{t}{{t}_{0}} when 0\le t\le {t}_{0}\\ \pi \frac{t-{t}_{0}+{t}_{r}}{{t}_{r}} when {t}_{0}\le t\le {t}_{0}+{t}_{r}\\ 0 when t\ge {t}_{0}+{t}_{r}\end{array}\right.$$

4 $${t}_{r}=ml+b$$

$m, b$ are constants that specify the shape of the linear relaxation duration and sarcomere length relaxation.${t}_{0}$ is time to reach peak tension after the initiation of active tension.

5 $$ECa_{50} = \frac{{\left( {Ca_{0} } \right)_{max} }}{{\sqrt {\exp \left[ {B\left( {l - l_{0} } \right)} \right] - 1} }};\;\;l = l_{R} \sqrt {2E_{ff} + 1}$$

where: ${E}_{ff}$ = Lagrangian strain in the fiber direction; $B$ is a constant that specifies the shape of the peak isometric tension-sarcomere length relation; ${l}_{0}$ is the sarcomere length that does not produce active stress; ${l}_{R}$ is the sarcomere length with the stress-free condition; ${\left(C{a}_{0}\right)}_{max}$ is the maximum peak intracellular calcium concentration.

The myocardial stress at the tissue level was the sum of active and passive stresses. In terms of loads and boundary conditions, in the LV-only model, blood pressure was applied to the endocardial surface during diastole, and a lumped parameter approach was used to simulate the arterial system. The results for the LV-only model pertain to the diastolic phase of cardiac cycle, and for the four-chamber model, the full cardiac cycle was simulated.

### Deep learning models

The mechanical properties of the four-chamber model and the LV-only model were varied to obtain the training and test data. The four-chamber model was used to study active mechanical properties, and the LV-only model was used to study passive material properties. The features for the models were obtained using a design of experiments (DOE) as explained in our previous report^10^.

The features in the four-chamber model and their ranges are as follows: 0.0015 < ${l}_{0}$ < 0.0028, 0.075 < ${t}_{0}$ < 0.25, 0.65 < ${T}_{max}$ < 1.9, for RV and LV tissues. Since pressure and volume were time-dependent, time was another feature in the four-chamber model. Also, when pressure was the output, volume was a feature and when volume was the output, pressure was a feature (number of features = 8). In total there were 80 four-chamber FE models. We used 6 FE models for the test set and 74 FE models for training and validation sets. In an additional analysis, we used 3 FE models for the test set and 77 FE models for training and validation sets. In both cases, the validation set comprised 10% of the sum of training and validation sets. Each FE model had 401 time points, which means there were 30,877 (= 401 × 77) and 1203 (= 401 × 3) data points for training and test, respectively. For each FE model, 401 data points were not independent, as they were from same model^15^.

The features and their range in the LV-only models were as follows: 0.387e−4 < $a$ < 9.881e−3, 0.005e−1 < ${a}_{f}$ < 49.901e−3, 9.1e−05 < ${a}_{s}$ < 6.986e−3, 4.4e−05 < ${a}_{fs}$ < 3.952e−3 MPa. Also, in these models, the stress data were calculated at each finite element centroid. As such, 3-dimensional coordinates of element centroids were also features of the model (number of features = 7). Together, the training and validation sets comprised 104 FE models and the test set consisted of 20 FE models. The validation FE models comprised 10% of the sum of training and validation sets. There were 576 elements in each FE model. Therefore, there were 59,904 (= 104 × 576) and 11,520 (= 20 × 576) data points for training and test, respectively. The training and test data for each FE model were not independent^15^.

**Figure 1:**
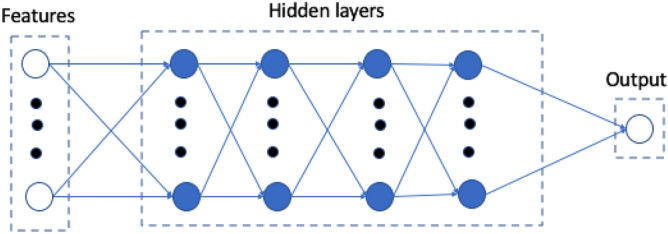
The feed forward DL model used for prediction of LV volume, pressure and stress.

A simple feed-forward DL network was used, but for LV pressure and volume the model structure was different from the model used for stress prediction (Fig. 1). The feed-forward DL network was composed of material properties as the features, 3 hidden layers and the outputs layer (Fig. 1). Each hidden layer had 128 neurons for stress predictions, and 20 neurons for pressure and volume predictions. There were 9 layers in the stress prediction model, and 5 layers in pressure and volume prediction models. PyTorch version 1.6.0 was used as the training library (http://pytorch.org). We used 200 and 2000 epochs for four-chamber and LV-only models, respectively, and the learning rate was 0.001. We used mean absolute error (MAE) as the loss function, and adaptive moment estimation (Adam)^16^ method as the optimization method. These hyper parameters were selected based on error analysis as explained in the next section. To avoid overfitting, we used early stopping. In particular, the optimized parameters were related to the epoch with minimum validation error.

As the heart functions in a sequential way, we used RNN with long short term (LSTM) to predict LV mechanics^17–20^. LSTM models have been developed to resolve the vanishing gradient problem in RNN whereby the information gradient becomes smaller as the number of time steps increases.

For estimation of LV mechanics, our RNN model assumes each LV FE model (with a new set of mechanical properties) is a sequence of the same FE model with another (previous) set of mechanical properties. In this context, previous refers to the FE model with last set of mechanical properties. Moreover, in each FE model, pressure and volume are sequences of previous time points and endocardial stresses at each element centroid are sequences of previous element centroid stresses. In this context, previous refers to element centroids with labels considered prior to the current element label.

**Figure 2:**
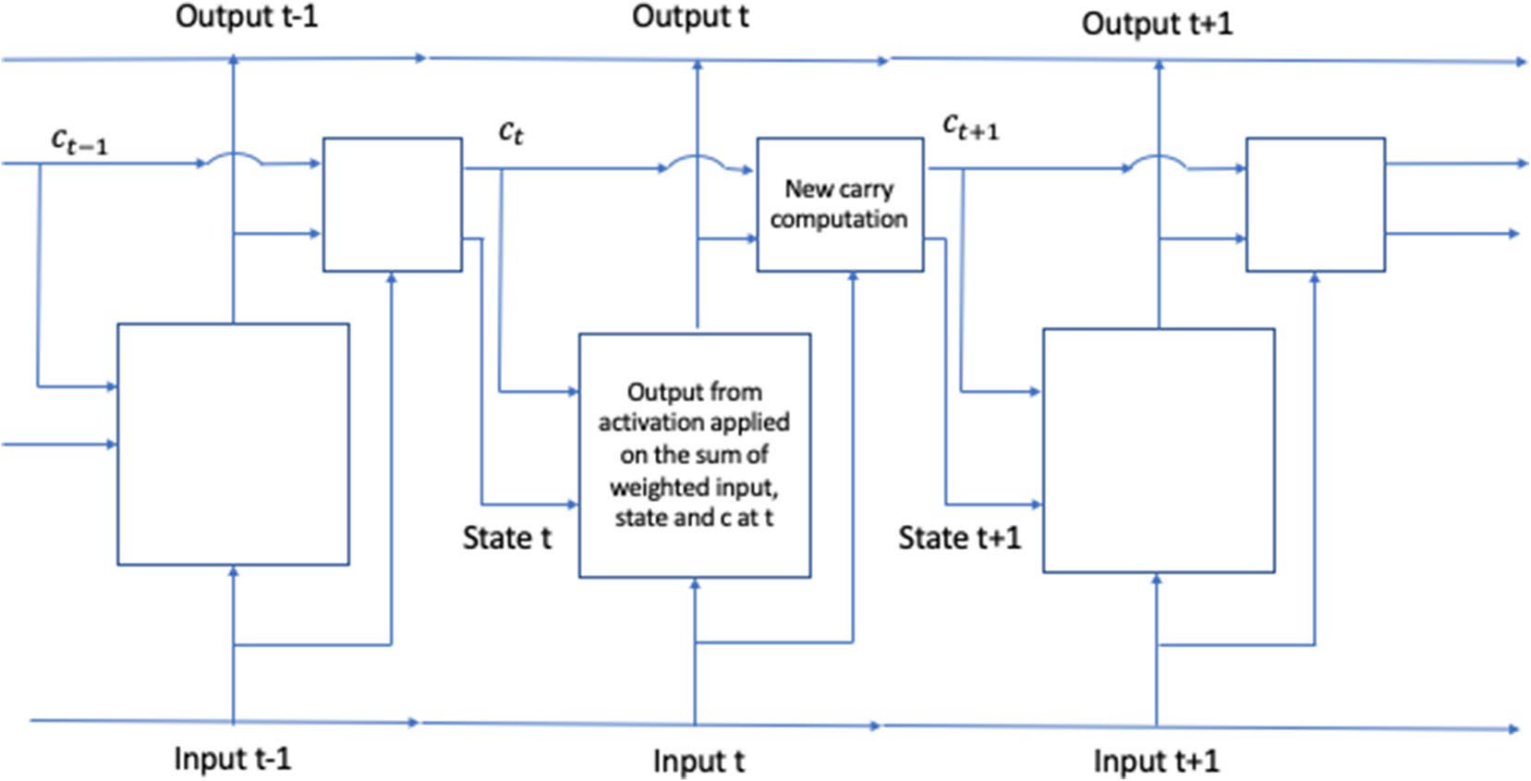
We used RNN with LSTM^17–20^. The LV pressure and volume from 20 previous time steps and active mechanical properties from current time step were used to predict current LV pressure and volume. Also, the LV endocardium stresses from 20 previous elements as well as passive mechanical properties from current time step were used to predict LV endocardium stresses at current time step. Here c represents carry state^19^.

The general workflow of our RNN model uses mechanical properties and LV output (pressure, volume or stress, Fig. 2). The LSTM portion of the model consisted of 32 LSTM units. We used 20 time points as the history for prediction of the current time step^19,20^. Similar to feed-forward models, for LV pressure and volume we had 77 and 3 models for training and testing, respectively, and for LV stress predictions we had 104 and 20 FE models for training and testing, respectively. The training and test data were divided into batches with a size of 401 for LV pressure and volume, and 576 for LV stresses, respectively. The RNN was trained with 1000 epochs and the epoch with lowest training error was used for testing model performance. This model was developed in TensorFlow version 2.2.0.

All the DL computations were performed on Google Collaboratory with Graphics Processing Units (GPUs).

### Error analysis

We evaluated the feed-forward DL predictions error by MAE calculated as follows:

6 $$MAE= {\frac{\sum_{i=1}^{n}\left|{y}_{i}-{\widehat{y}}_{i}\right|}{n}}$$

where $y$ and $\widehat{y}$ are the FE and ML outputs, and $i$ pertains to either each time point (pressure and volume predictions) or element centroids (stress predictions), and $n$ is the number of datapoints/elements in each test model. Because the predicted value for pressure (or volume) at each time point was not independent from other time points in each FE model, we calculated the average error for all test FE models. A similar calculation was performed for stress predictions as follows:

$${MAE}_{Average}=\frac{\sum_{1}^{m}MAE}{m}$$

where $m$ is number of test models.

## Results

**Figure 3:**
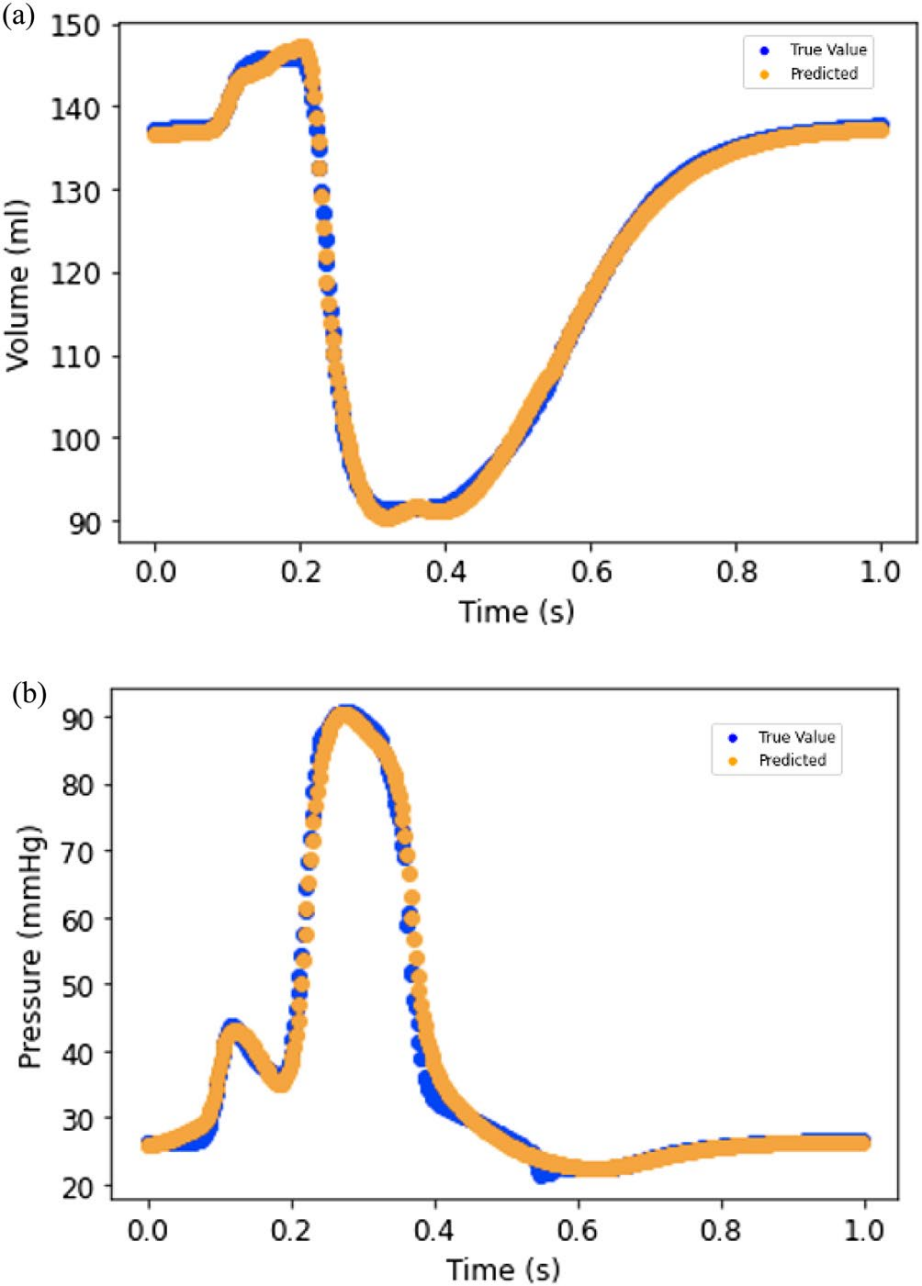
(**a**) LV volume from FE (blue) and feed forward DL (orange); (**b**) LV pressure from FE (blue) and feed forward DL (orange).

**Figure 4:**
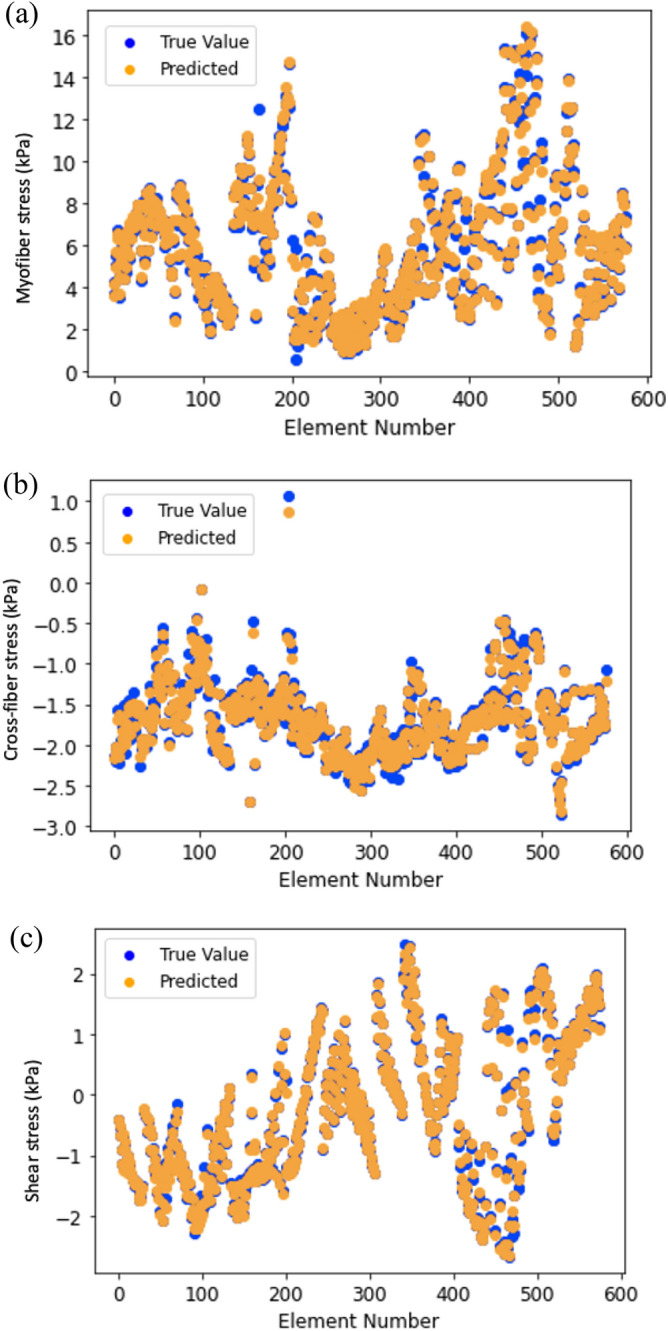
(**a**) Myofiber stress from FE (blue) and feed forward DL (orange); (**b**) Cross-fiber stress from FE (blue) and feed forward DL (orange); (**c**) Shear stress from FE (blue) and feed forward DL (orange).

The feed-forward DL-predicted volume and pressures in the four-chamber model were in close agreement with FE data (Figs. 3, 4). The feed-forward DL model was capable of producing the shape of the volume and pressure waves. For volume and pressure predictions, the average MAE was relatively small (Table 1).

**Table 1:** The error results for LV stress, volume and pressure predictions.

	MAE ± SD	
	DL	XGBoost
Myofiber stress (kPa)	0.179 ± 0.050	0.334 ± 0.228
Cross-fiber stress (kPa)	0.049 ± 0.017	0.075 ± 0.024
Shear stress (kPa)	0.039 ± 0.011	0.050 ± 0.032
Volume (ml)^a^	0.687 ± 0.165	1.734 ± 0.584
Pressure (mmHg)^a^	0.923 ± 0.285	1.544 ± 0.298
Volume (ml)^b^	1.599 ± 1.227	–
Pressure (mmHg)^b^	1.257 ± 0.488	–

Also, DL results are compared with XGBoost from our previous study^10^.

^a^Using 3 test FE models.

^b^Using 6 test FE models.

Similarly, the feed forward DL-predicted stresses were noticeably close to FE results with a pattern of stress results that was noticeably similar to FE results (Fig. 4). The average MSE was relatively small (Table 1). The predictions for all three components of stress were similarly in good agreement with FE data.

**Figure 5:**
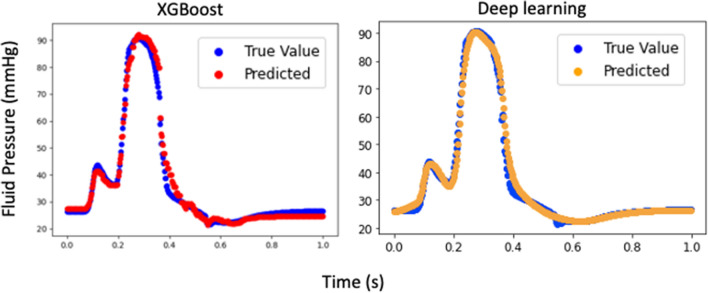
Comparison between XGBoost and feed forward DL. The XGBoost result is reproduced from our previous paper^10^.

The runtime for feed-forward DL predictions were much shorter than that for FE models. The training time for same number of epochs was different for pressure and volume predictions. Also, the training time for different components of stress was different. For example, the runtime for stress predictions is nearly 20 min, but the equivalent results were computed using feed- forward DL on the order of seconds. Also, the feed-forward DL results were comparable to our eXtreme Gradient Boosting (XGboost) results^10^ (Table 1 and Fig. 5).

**Figure 6:**
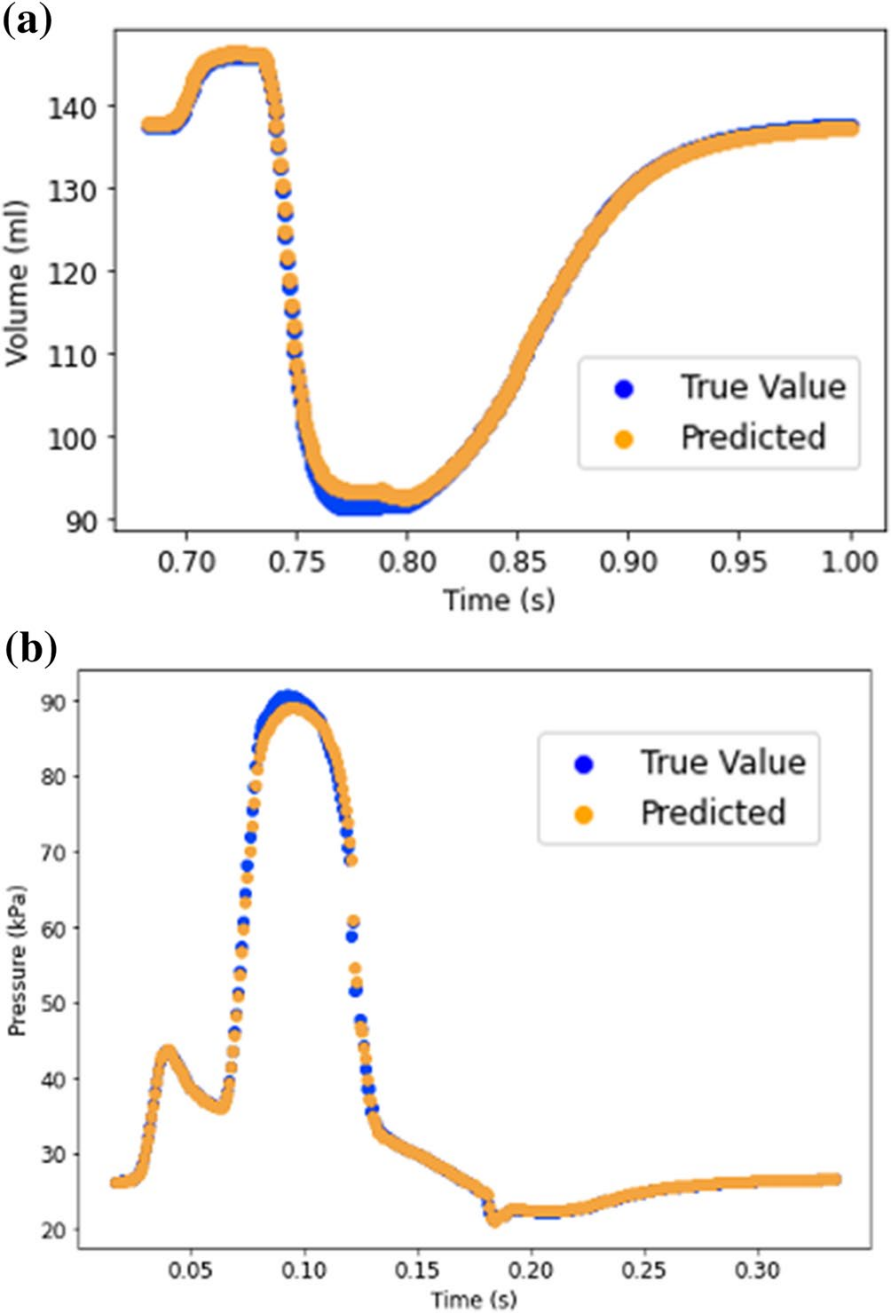
Prediction of LV (**a**) volume and (**b**) pressure using RNN with LSTM. Each cardiac cycle (FE mode) was assumed as a sequence of previous cardiac cycle (FE model). Moreover, at each time point, data from past 20 time points were used to predict current time pressure or volume.

**Figure 7:**
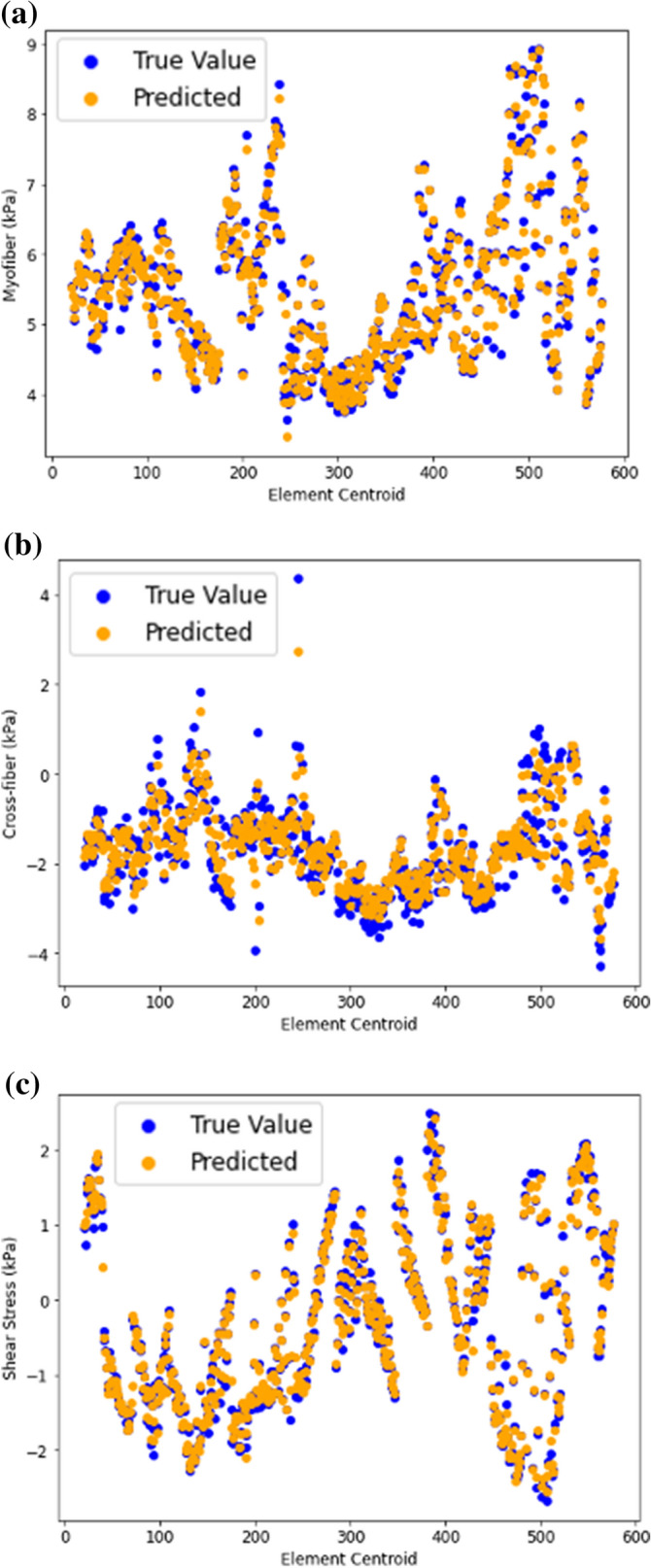
Endocardial (**a**) myofiber stress, (**b**) cross-fiber stress and (**c**) shear stresses predicted using RNN with LSTM. Each FE model was assumed as a sequence of previous FE model. Moreover, at each element centroid data from 20 previous elements were used.

The results from RNN were similar to those for FE models (Figs. 6 and 7). The LV pressure and volume estimated using RNN were noticeably close to FE results. Also, RNN models estimated LV endocardial stresses close to FE predictions. As shown in the sample results (Figs. 6 and 7), the RNN could estimate details of LV data. The LV pressure and volume at each time step and endocardial stresses at each element centroid were noticeably close to corresponding FE data. Moreover, the estimated LV pressure, volume and stresses were smooth. The differences between RNN and FE results were more noticeable at stress outliers where the stress value was notably higher compared to other element centroids (Fig. 7b).

**Figure 8:**
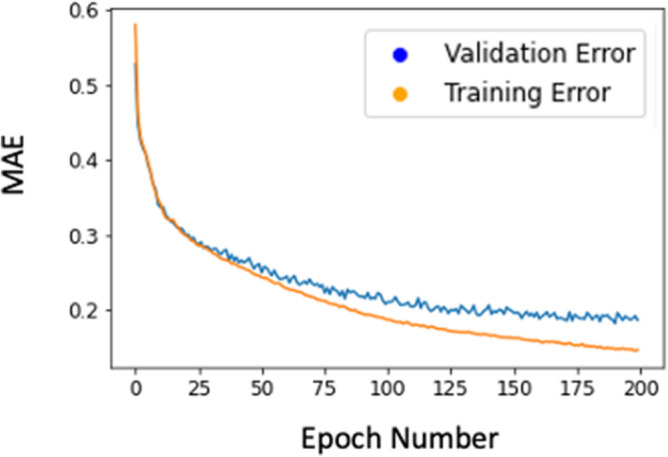
The training and validation errors for the cross-fiber direction. Other outputs had similar patterns.

Hyper parameter tuning was important for both feed-forward and RNN models. For feed-forward models we selected the optimized solution based on minimized validation loss (Fig. 8), and for RNN we selected optimized solution based on minimized training loss. The training and validation losses were relatively high in initial epochs, but became smaller as more epochs were performed. After some epochs the validation error started to increase and continued to do so. The optimized parameters based on minimized losses provided better results than other epochs with higher losses. The learning rate, number of layers, number of neurons, type of activation function, loss function and optimization method needed to be appropriately manipulated to find reasonable estimations.

## Discussion

Although we have used FE modeling extensively to study LV mechanics^4,5,21,22^, the runtimes for these simulations are too long for real-time and/or clinical applications. DL has been used in studies of cardiovascular biomechanics^6,7^. In this study, our goal was to investigate applicability of DL in predicting ventricular mechanics. We introduced application of both feed-forward DL and RNN in LV biomechanics, namely stress, pressure and volume predictions. Our results indicate that DL models can predict ventricular biomechanics with high accuracy and speed. In terms of accuracy, MAE for the feed-forward DL was relatively low for pressure, volume and stress predictions (Table 1). In terms of speed, the DL results were obtained in a matter of seconds (after training) whereas equivalent FE results required several CPU hours (pressure and volume) or 20 min of runtime (stress)^10^. These results suggest that using neural networks is an option when the mechanical behavior of the heart is required in real-time.

In a clinical workflow, the ML approach has other advantages over using the long-running FE models directly for decision making. Creating an FE model is prone to error because of the large number of inputs required, whereas an ML-based model requires a reduced number of inputs. In our experience, the FE modeling error rate matches that of transcribing errors^23^, around one error every 30 entries. In addition, the expense and long simulation times required for FE modeling make it impractical to practice the double-entry method for modeling or check the results sequentially in a clinical workflow. Another advantage of ML models is that they can verify the FE simulations on which they are based. Erroneous FE simulations as part of a DOE study typically stand out immediately once they are trained into an ML model.

Our RNN model showed that LSTM units can be used to predict the LV pressure, volume and endocardial stresses with relatively high agreement with FE ground truth data. In particular, when we fed 20 previous time points/element stresses to the RNN, this model predicted the LV data in relatively close agreement with the ground truth values (Figs. 6 and 7). In both the feed-forward DL models (Figs. 3 and 4) and in our XGBoost model (Fig. 5)^10^ time was included as a feature for predictions, but the history of the LV was not. In the RNN (Figs. 6 and 7), the LV behavior was predicted from 20 past time points/element stresses. In comparison to other ML models that we have used to predict LV mechanics, RNN showed that inclusion of time history behavior plays an important role. However, for RNN, we need to have data from previous time points/elements in addition to previous relevant FE models.

In this paper we used both material properties from current time points (or elements) as well as information from previous time points (or elements) to predict LV pressure and volume (or stresses) using RNN. Two other possibilities were to (1) just use data from previous time points to make predictions; and (2) make predictions for several time steps instead of only at the current time. For example, if the goal is to predict LV pressure, using the former approach, only pressure data from previous time points can be used to make predictions. On the other hand, instead of making the prediction for the current time, LV pressure can be predicted for the next 10 time points (as an example). Results from these approaches would be different from our results, as the amount of information used to make predictions would be different. A future direction could be to compare these different scenarios and select the best approach based on application requirements.

To assess the performance of the DL models, we performed the volume and pressure computations with two different numbers of test FE models. First, we used 6 test FE models. One aspect of this study was to compare DL and XGBoost predictions from our previous report^10^. Since in our previous report^10^, there were 3 FE test models, we performed an additional analysis with 3 test FE models. With data from 6 test FE models, errors were larger (Table 1), but the larger errors could be due to smaller number of training FE models as we had to decrease the number of training FE models to increase the number of test FE models. As a future direction, more FE models can be created to increase the size of the test set. The number of test sets for the stress predictions was over 16% of data, similar to our XGBoost results^10^. The test samples used in this paper did not show noticeable overfitting in the results as the ML results for test data were close to FE results (Table 1, Figs. 3–7). However, incorporating more datasets will help to improve the predictions.

In our study, DL was used to predict LV biomechanical characteristics from material properties as input features, but a more clinically relevant problem to address would be to predict mechanical properties of LV from measurable information such as pressure, volume and strain data. In other words, a DL model could be used to calibrate the mechanical properties of the LV for IoT and hand-held devices (such as echocardiography) for diagnostic and preventive purposes. This application is particularly important in patients with HF because the myocardial mechanical properties are related to HF development^4^. Also, other parameters can be considered as features of the model, including the geometry of the LV, electrical characteristics of myocardium, heart rate, and presence of LVADs.

The LV flow and pressure can be measured experimentally and used as a reference for DL predictions. Since the homeostatic stress level is essential to the function of the LV^24^, a correlation can be made between DL stress predictions and myocardial function. Our methodology can be used for applications that require real-time data such as surgical or interventional procedure planning to avoid time-consuming FE models.

We used active and passive mechanical properties to create in-silico data for pressure/volume and stress predictions, respectively. We assumed a homogeneous material behavior for the myocardium, which means the same material properties were used throughout the LV myocardium. Future studies should include creation of datasets based on heterogenous properties. In healthy myocardium, experimental studies reported variations of material characteristics of the myocardium^25,26^. In patients with myocardial infarction, the mechanical properties of the infarcted region differ from the healthy region^27,28^. Also, development of heart failure with preserved ejection fraction could be related to transmural variation in myocardial deformations and mechanical properties^4^. Moreover, the loads and boundary conditions could be different in the dataset. For example, LVADs alter the loads and boundary conditions of the heart^22^, effects of which, can be predicted in real time, using our DL methodology.
